# How Do Brown Seaweeds Work on Biomarkers of Dyslipidemia? A Systematic Review with Meta-Analysis and Meta-Regression

**DOI:** 10.3390/md21040220

**Published:** 2023-03-30

**Authors:** Dayeon Shin, Sung Ryul Shim, Yueying Wu, Gayeon Hong, Hyunyu Jeon, Choong-Gon Kim, Kyung Ju Lee

**Affiliations:** 1 Department of Food and Nutrition, Inha University, Incheon 22212, Republic of Korea; 2 Department of Health and Medical Informatics, Kyungnam University College of Health Sciences, Changwon-si 51767, Republic of Korea; 3 Department of Biomedical Informatics, College of Medicine, Konyang University, 158 Gwanjeodong-ro, Seo-gu, Daejeon 35365, Republic of Korea; 4 Marine Ecosystem Research Center, Korea Institute of Ocean Science and Technology, Busan 49111, Republic of Korea; 5 Department of Women’s Rehabilitation, National Rehabilitation Center, Seoul 01022, Republic of Korea

**Keywords:** brown seaweed, dyslipidemia, marine, health benefits

## Abstract

Dyslipidemia is a common chronic disease that increases the risk of cardiovascular disease. Diet plays an important role in the development of dyslipidemia. As people pay increased attention to healthy eating habits, brown seaweed consumption is increasing, particularly in East Asian countries. The association between dyslipidemia and brown seaweed consumption has been previously demonstrated. We searched for keywords associated with brown seaweed and dyslipidemia in electronic databases such as PubMed, Embase, and Cochrane. Heterogeneity was estimated using the I^2^ statistic. The 95% confidence interval (CI) of the forest plot and heterogeneity were confirmed using meta-ANOVA and meta-regression. Funnel plots and publication bias statistical tests were used to determine publication bias. Statistical significance was set at *p* < 0.05. In this meta-analysis, we found that brown seaweed intake significantly decreased the levels of total cholesterol (mean difference (MD): −3.001; 95% CI: −5.770, −0.232) and low-density lipoprotein (LDL) cholesterol (MD: −6.519; 95% CI: −12.884, −0.154); nevertheless, the statistically significant association of brown seaweed intake with high-density lipoprotein (HDL) cholesterol and triglycerides were not observed in our study (MD: 0.889; 95% CI: −0.558, 2.335 and MD: 8.515; 95% CI: −19.354, 36.383). Our study demonstrated that brown seaweed and its extracts decreased total cholesterol and LDL cholesterol levels. The use of brown seaweeds may be a promising strategy to reduce the risk of dyslipidemia. Future studies involving a larger population are warranted to investigate the dose–response association of brown seaweed consumption with dyslipidemia.

## 1. Introduction

Non-communicable diseases (NCDs), which continue to increase, account for 74% of deaths worldwide [1]. People in low- and middle-income countries with NCDs account for more than three-quarters of NCD deaths worldwide [1]. Major types of NCDs include cardiovascular diseases, cancer, chronic respiratory diseases, and diabetes [1]. One of the contributing risk factors for noncommunicable diseases is increased metabolic changes, such as dyslipidemia. 

The prevalence of dyslipidemia in Korean adults was 38.4% in 2018 according to the Korea National Health and Nutrition Examination Survey [2]. In 2020, the prevalence of hypercholesterolemia in Korean adults was 23.7% [3]. Hypercholesterolemia, the most common form of dyslipidemia, reduces the arterial blood flow and capillary density, resulting in capillary endothelial cell death and impaired left ventricular function [4]. Dyslipidemia is a common primary risk factor for cardiovascular diseases, atherosclerosis, and stroke [5]. Cardiovascular disease is the leading cause of death worldwide and dyslipidemia is a fundamental risk factor associated with cardiovascular disease [6]. Therefore, it is important to reduce dyslipidemia to prevent cardiovascular diseases.

The identified risk factors for dyslipidemia include obesity, diabetes, hypertension, smoking status, advanced age, and lifestyle changes [7,8,9]. Moreover, unhealthy dietary habits, such as excessive intake of saturated and trans fats, refined carbohydrates, and sugar, can increase the risk of dyslipidemia [10,11]. Diet is a modifiable factor that can be controlled easily. Among dietary factors, seaweed consumption is inversely associated with the risk of dyslipidemia in the Korean population [12,13]. Furthermore, previous findings indicated that Korean adults with type 2 diabetes who were administered seaweed exhibited reduced blood glucose and lipid levels and increased antioxidant enzyme activities [13]. High cholesterol and triglycerides levels are associated with an increased risk of atherosclerotic cardiovascular disease (CVD) [14], stroke, and atherosclerosis [5]. The desirable levels per guideline are as follows: (1) total cholesterol < 200 mg/dL, (2) low-density lipoprotein (LDL) cholesterol < 100 mg/dL, (3) high-density lipoprotein (HDL) cholesterol > 60 mg/dL, and (4) triglyceride <150 mg/dL [14,15]. In the seaweed supplement group, after four weeks from baseline, serum triglyceride concentration was significantly decreased (baseline: 171.2 ± 20.7 vs. 4 weeks: 118.8 ± 17.6) and HDL cholesterol concentration was significantly increased (baseline: 37.1 ± 3.2 vs. 4 weeks: 44.6 ± 2.9, *p* < 0.05) [13]. Seaweeds are rich in various nutrients and health-promoting compounds [16], such as polyphenols (alginates, fucose-containing sulfated polysaccharides, and laminarins) and terpenoids (carotenoids and sterols), which can reduce the risk of dyslipidemia [17,18]. 

Seaweeds are categorized into three varieties: red, brown, and green [19]. Brown seaweeds, particularly kombu (*Laminaria japonica*), wakame (*Hijikia fusiforme*), and hijiki (*Undaria pinnatifida*), are popular cooking ingredients in East Asian countries [20,21]. Previous findings indicated that seaweed intake is inversely associated with ischemic heart disease in Japanese men and women [22]. The authors stated that fucoidan in brown seaweed may regulate the metabolism of serum lipids by increasing lipoprotein lipase activity [23], which may have reduced the levels of triglycerides and LDL cholesterol.

In an animal experiment, supplementation with fucoidan resulted in a significant reduction in the levels of triglycerides, total cholesterol, non-HDL cholesterol (total cholesterol minus HDL cholesterol), blood lipids, and glucose; total cholesterol and triglyceride levels decreased by 24.3% and 32.4%, respectively (*P* < 0.05) [23]. In a study on the potential of various seaweed extracts to lower lipid levels in rats, the brown seaweeds *S. binderi* and *J. laminarioides* reduced triglyceride levels in hyperlipidemic rats fed a high-fat diet by 31.6% and 33%, respectively [24]. The number of systematic reviews on the association between brown seaweed and dyslipidemia in humans is fewer than that in animals, and there are even fewer meta-analyses relating to humans. Moreover, the therapeutic effects of brown seaweed on dyslipidemia remain unclear. Based on the various findings of human intervention studies, a comprehensive analysis of the evidence is necessary. Thus, we aimed to perform a systematic review of meta-analyses on brown seaweed consumption and dyslipidemia and evaluate the factors contributing to dyslipidemia using meta-regression.

## 2. Materials and Methods

### 2.1. Data sources and Search Strategy

We investigated online bibliographic databases such as PubMed, Embase, and Cochrane using the following keywords: “seaweed”, “brown seaweed”, “brown seaweed extract”, “phaeophyta”, “brown algae”, “phaeophyceae”, “brown algae extract”, “fucoidan”, “ventol”, “dioxinodehydroecko”, “2,7-phloroglucinol-6,6′-bieckol”, “*Ecklonia cava*”, “Undaria”, “Wakame”, and “fucoxanthin.” We also searched for “cholesterol”, “epicholesterol”, “HDL cholesterol”, “high density lipoprotein cholesterol”, “dyslipidemias”, “total cholesterol”, and “triglycerides” as terms associated with dyslipidemia (Table S1). We retrieved additional study reports from 2021 to 2022 that were not identified in the database search. The first screening was independently performed by two researchers (Shin D and Shim SR) based on the title and abstract to identify ineligible studies. Potentially relevant studies were also reviewed. 

### 2.2. Eligibility Criteria

The present systematic review and meta-analysis were conducted according to the Population, Intervention, Comparison, and Outcome (PICO) databases (Table 1). The final inclusion of each article was determined by all the investigators through evaluation and discussion. To ensure the integrity of the meta-analysis and absence of overlapping data, the references and data for each included study were cross-checked. 

### 2.3. Data Extraction and Measurement Outcomes

The data were compiled by creating a coding table. The study, intervention, control, study duration, subject description, N, mean, and standard deviation were calculated. Subsequently, N, mean, and standard deviation were entered based on the indicators compared before, after, and associated with metabolic syndrome. In addition, for fewer than three indicators, the case was considered difficult to compare and excluded. Primary outcomes were total cholesterol, HDL cholesterol, LDL cholesterol, and triglyceride levels. 

### 2.4. Data and Statistical Analyses

Mean differences with 95% confidence intervals (CIs) were used to analyze all-inclusive mean differences. Statistical significance was set at *P* < 0.05. Heterogeneity was estimated using the I^2^ statistic. The 95% confidence interval (CI) of the forest plot was confirmed, and heterogeneity was confirmed using meta-ANOVA and meta-regression. Funnel plots and statistical tests were used to determine the publication bias. If it was distributed on both sides within the top of the funnel, there was no risk of bias. Further, R software (version 4.2.2.) (R Foundation for Statistical Computing, Vienna, Austria) was used for the data analysis. Two researchers independently performed quality assessments according to the Cochrane Risk of Bias Tool (RoB 2.0) [25,26]. Quality was evaluated as low, with some concerns, or high using the tool algorithm. Subsequently, the two researchers discussed the domains using different assessments and reached an agreement.

### 2.5. Quality Assessment

The risk of bias and methodological quality were assessed using the Cochrane Collaboration RoB 2.0 tool which includes five domains and an overall judgment [25,26]. Each domain’s judgment influences the overall risk of judgment bias of the assessment outcome [25]. We evaluated five parameters: (1) the randomization process, (2) deviations from the intended interventions, (3) missing outcome data, (4) measurement of the outcome, and (5) selection of the reported result. Each domain was assigned a risk-of-bias rating of high, low, or unclear. The overall risk of bias was considered to be low if all domains were rated as “Low”, some concerns if even one domain was rated as “Some concerns”, and high risk if even one domain was rated as “High”, or more than two domains were rated as “Some concerns”.

## 3. Results

### 3.1. Study Selection

**Figure 1:**
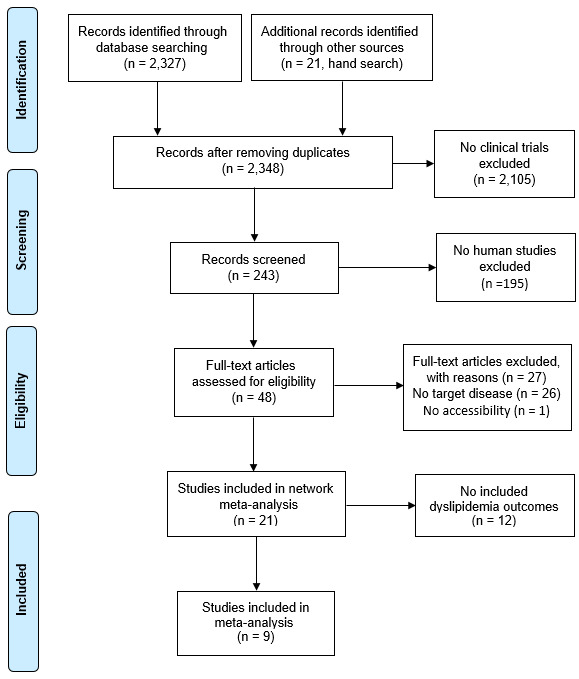
Preferred reporting items for systematic reviews and meta-analyses (PRISMA) flow diagram of studies included in the selection process.

A total of 2327 study reports were searched: 17 in PubMed, 2305 in Embase, and 5 in Cochrane. Subsequently, 21 studies were included and additional records were identified through manual search. Among them, 195 nonhuman studies were excluded, leaving 2105 papers that were not included as clinical trials. In addition, 26 papers without the target diseases and 1 unavailable paper were excluded. Nine studies were chosen after eliminating twelve studies that did not include indicators of dyslipidemia (Figure 1).

The present systematic review included nine studies, including two randomized, double-blind, placebo-controlled, crossover design studies; three randomized, double-blind, placebo-controlled, parallel design studies; one randomized, double-blind, placebo-controlled intervention study; one randomized, double-blind, parallel-group, placebo-controlled pilot study; and two randomized, single-blind, controlled clinical trials. The general characteristics of the included studies are summarized in Table 2.

The age of the participants ranged from 12 to 72 years. Three studies [27,28,29] were conducted on healthy participants (*n* = 103). Three studies [13,30,31] involving participants with type 2 diabetes mellitus (T2DM; *n* = 212) were included in the meta-analysis. Vodouhé et al. (1987) [32] investigated the effects of obesity on patients likely to develop T2DM. Hernández-Corona et al. (2014) [33] examined obese participants (*n* = 25), and Mikami et al. (2017) [34] investigated the effects in participants with normal weight or obesity.

Nine studies [13,27,28,29,30,31,32,33,34] investigated the effect of seaweed extracts on dyslipidemia biomarkers. The most common extracts were *Ascophyllum nodosum/Fucus vesiculosus* (*n =* 2), fucoidan (*n =* 3), *Laminaria japonica/alginate* (*n =* 1), *Laminaria japonica* (*n =* 1), calcium alginate (*n =* 1), and fucoxanthin (*n =* 1). The dosage differed among the studies (175 mg to 48 g) according to the type of seaweed.

**Table 2:** Characteristics of the included studies.

Study	Geographic Location	Sample Size	Age	Type of Brown Seaweed	Dosage	Duration	Measured Outcomes
Kim 2008 [13]	Korea	20	40–70 years	*Laminaria japonica* (Sea tangle)	48 g	4 weeks and 2 h	↑ HDL-Cho, ↓ T-Cho, LDL-Cho, TG
Hernandez-Corona 2014 [33]	Mexico	25	30–60 years	(No name given) Fucoidan extract	500 mg	3 months and 2 h	↓ T-Cho, LDL-Cho
Mikami 2017 [34]	Japan	40	30–77 years	Fucoxanthin	220 mg	8 weeks	↑ HDL-Cho, ↓ LDL-Cho
Kato 2018 [27]	Japan	30	≥20 years	(No name given) Calcium alginate extract	Ca-Alg 5%, 3.2 g Ca-Alg 8%, 5.0 g	2 h	↓ T-Cho, LDL-Cho, TG
Derosa 2019 [31]	Italy	164	>18 years	*Ascophyllum nodosum*, *Fucus vesiculosus*	-	6 months	↑ HDL-Cho, ↓ T-Cho, LDL-Cho, TG
Sakai 2019 [30]	Japan	28	59.10 ± 13.24 years	Mozuku fucoidan extract	1620 mg	3 months	↓ T-Cho
Aoe 2021 [29]	Japan	48	20–59 years	*Laminaria japonica*/alginate	6 g kelp powder/d 3.3 g alginate/d	2 months	↓ T-Cho, LDL-Cho
Tomori 2021 [28]	Japan	40	47.0 ± 7.6 years	Fucoidan derived from *Okinawa mozuku* (South product, Uruma, Japan)	3.0 g	16 weeks	↑ HDL-Cho
Vodouhe 2022 [32]	Canada	56	18–70 years	*Ascophyllum nodosum* and *Fucus vesiculosus* Extract	500 mg	3 months	↓T-Cho, LDL-Cho

T-Cho, total cholesterol; HDL-Cho, HDL cholesterol; LDL-Cho, LDL cholesterol; TG, triglyceride.

### 3.2. Quality Assessment

We used RoB 2.0, a tool for measuring bias risk, to critically evaluate the nine selected studies. All included studies were randomized. Two were randomized, double-blind, placebo-controlled, and crossover studies, and three were randomized, double-blind, placebo-controlled, and parallel-design studies. One was a randomized, double-blind, placebo-controlled intervention study; one was a randomized, double-blind, parallel-group, placebo-controlled pilot study; and the other two were randomized, single-blind, controlled clinical trials. The overall quality of the studies was estimated to be high risk for two studies and low risk for seven. This low risk was due to exposure to the uncontrolled environment in the laboratory.

### 3.3. Outcomes

#### 3.3.1. Total Cholesterol

**Figure 2:**
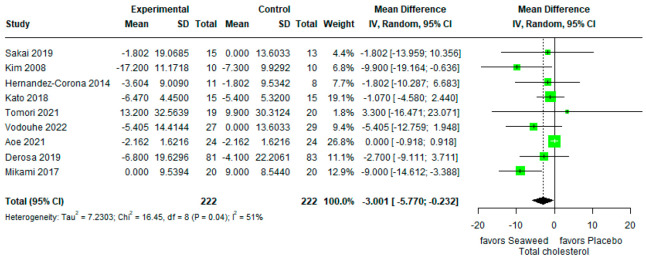
Forest plot of the mean difference between treatment and placebo groups indicating the effect of brown seaweed supplementation on total cholesterol.

The effects of brown seaweed on total cholesterol in this meta-analysis are shown in Figure 2. A statistically significant decrease in total cholesterol levels between the control and experimental groups was observed (mean difference, −3.001 [95% CI, −5.770, −0.232]). This suggests that brown seaweed significantly reduces total cholesterol levels in participants with T2DM (Sakai [30], Kim [13], Derosa [31]), participants with obesity (Corona [33]), participants with obesity/pre-diabetes (Vodouhè [32]), and participants with normal weight/obesity (Mikami [34]). Heterogeneity of the pooled estimates was high (I^2^ = 51%).

#### 3.3.2. HDL Cholesterol

**Figure 3:**
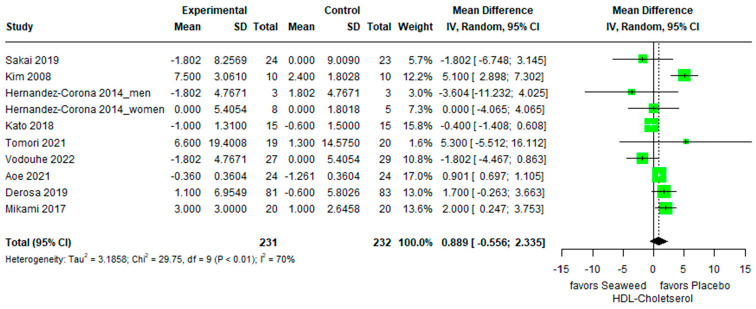
Forest plot of the mean difference between treatment and placebo groups for the effect of brown seaweed supplementation on HDL cholesterol.

The effects of brown seaweed on HDL cholesterol levels are shown in Figure 3. Notably, HDL cholesterol levels showed an upward trend, but the difference was not significant between the control and experimental groups (mean difference 0.889 [95% CI: 0.558, 2.335]). Brown seaweed promotes HDL cholesterol secretion in patients with T2DM, healthy individuals, and participants with normal weight or obesity. However, these results were considered unreliable. Heterogeneity of the pooled estimates was high (I^2^ = 70%).

#### 3.3.3. LDL Cholesterol

**Figure 4:**
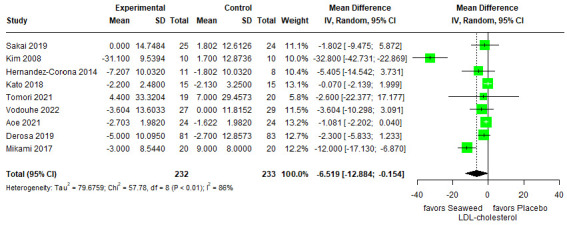
Forest plot of the mean difference between treatment and placebo groups indicating the effect of brown seaweed supplementation on LDL cholesterol.

The effects of brown seaweed on LDL cholesterol levels are shown in Figure 4. The differences between the control and experimental groups were statistically significant. The LDL cholesterol level in the experimental group was lower than that in the control group (mean difference, −6.519 [95% CI −12.884, −0.154]). This suggests that brown seaweed has been associated with decreased LDL cholesterol levels in participants with T2DM (Sakai [30], Kim [13], Derosa [31]), obesity/pre-diabetic (Vodouhè [32]), normal-weight/obesity (Mikami [34]), obesity (Corona [33]), and healthy participants (Kato [27], Tomori [28], Aoe [29]). Heterogeneity of the pooled estimates was high (I^2^ = 86%).

#### 3.3.4. Triglycerides

**Figure 5:**
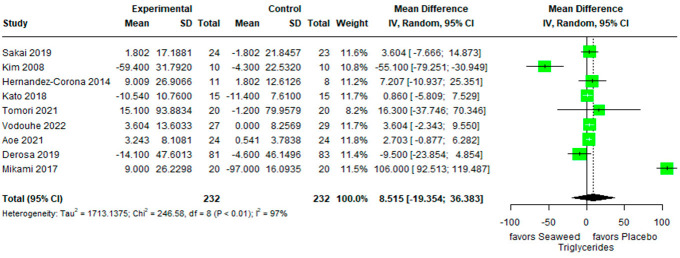
Forest plot of the mean difference between treatment and placebo groups indicating the effect of brown seaweed supplementation on triglycerides.

The effects of brown seaweed on triglycerides are shown in Figure 5. No significant difference between the experimental and control groups was observed (mean difference 8.515 [95% CI −19.354, 36.383]). Heterogeneity of the pooled estimates was high (I^2^ = 97%).

### 3.4. Meta-Regression

Table 3 outlines the results of subgroup and meta-regression analyses. In the meta-regression analysis, subgroup analyses by duration (greater and less than 3 months), race (Asian, Hispanic, and Caucasian), and female participation rate did not reveal statistically significant differences in the reduction in risk factors for dyslipidemia. Although not statistically significant, a higher female participation rate showed a decreasing tendency in total cholesterol (SMD: −13.141, 95% CI −42.559, 16.316) and LDL cholesterol levels (SMD: −14.172, 95% CI −70.258, 41.914).

### 3.5. Evidence

#### 3.5.1. Evidence of *Ascophyllum nodosum*/*Fucus vesiculosus*

Derosa et al. (2019) and Vodouhè et al. (2022) used *Fucus vesiculosus* and *Ascophyllum nodosum* extracts from brown seaweed [31,32]. They analyzed the effects of brown seaweed extracts of *Ascophyllum nodosum* and *Fucus vesiculosus* on the biomarkers of dyslipidemia, such as total cholesterol, HDL cholesterol, LDL cholesterol, and triglycerides, in individuals with obesity, pre-diabetes, and T2DM. Participants were administered either *Ascophyllum nodosum/Fucus vesiculosus* (500 mg/day) or placebo with personalized nutritional advice for controlled weight loss. Results of studies on *Ascophyllum nodosum/Fucus vesiculosus* extracts showed no significant reduction in the biomarkers of dyslipidemia. Vodouhè et al. (2022) demonstrated a decrease in total cholesterol, HDL cholesterol, and LDL cholesterol and an increase in triglycerides but reported no significant effects of *Ascophyllum nodosum/Fucus vesiculosus* extracts on obesity and pre-diabetes [32]. Derosa et al. (2019) investigated the association between dyslipidemia biomarkers and brown seaweed extracts of *Ascophyllum nodosum/Fucus vesiculosus* in patients with T2DM [31]. The results showed lower total cholesterol, LDL cholesterol, and triglyceride levels and increased HDL cholesterol levels, which were insignificant in participants with T2DM.

#### 3.5.2. Evidence of Fucoidan

Tomori et al. (2021), Sakai et al. (2019), and Hernández-Corona et al. (2014) evaluated the effect of fucoidan on dyslipidemia extract biomarkers, such as total cholesterol, HDL cholesterol, LDL cholesterol, and triglycerides in participants with T2DM, obesity, overweight, and healthy adults [28,30,33]. Furthermore, the effects of fucoidan on dyslipidemia biomarkers were evaluated. In these studies, participants in the fucoidan group consumed fucoidan extract (500 mg to 3.0 g) for a certain time period via oral administration or drinks, and participants in the control group were administered a placebo [28,30,33]. The appearance of the placebo beverage was similar to that of the fucoidan beverage. They investigated the effects of fucoidan extracts on biological indicators of dyslipidemia, such as total cholesterol, LDL cholesterol, and triglycerides, but reported no significant effects. Sakai et al. (2019) and Hernández-Corona et al. (2014) reported the outcomes of dyslipidemia biomarkers in participants with T2DM and obesity, indicating a reduction in total cholesterol, LDL cholesterol, and triglyceride levels, but reported no significant effect of the fucoidan extract [30,33]. A study by Tomori et al. (2021) on the effects of fucoidan indicated a decrease in total cholesterol, LDL cholesterol, and triglyceride levels and an increase in HDL cholesterol levels, but did not report significant results in healthy participants [28]. The results indicated that neither low nor high doses (500 mg to 3.0 g) of fucoidan extract had a significant effect on dyslipidemia biomarkers in participants with T2DM and obesity and in healthy adults.

#### 3.5.3. Evidence of *Laminaria japonica* and *Undaria pinnatifida*

*Laminaria japonica* was used to evaluate the effects on dyslipidemia biomarkers, such as total cholesterol, HDL cholesterol, LDL cholesterol, and triglycerides, in participants with T2DM and healthy participants [13,29]. The participants were randomized into a control group or a seaweed supplement group. The participants took a pill composed of the seaweed supplement *Laminaria japonica* thrice a day for four weeks. The total daily consumption of brown seaweeds was 48 g [13]. In another study, 30 tablets (10 tablets, three times/day) containing 6 g of kelp powder and 3.3 g of alginate were administered to the test group three times a day, and kelp-free powders were administered to the placebo group for eight weeks [29]. Kim et al. (2008) demonstrated that on consumption of the *Laminaria japonica* extract (dry powdered sea tangle and sea mustard) pills, the total cholesterol (MD −9.900, 95% CI −19.164 to −0.636), LDL cholesterol (MD 32.800, 95% CI −42.731 to −22.869), and triglyceride levels (MD −55.100, 95% CI −79.251 to −30.949) of participants in the seaweed supplement group were significantly lower compared with those in the control group [13].

#### 3.5.4. Evidence of Calcium Alginate

The effects of the calcium alginate extract on dyslipidemia biomarkers, such as total cholesterol, HDL cholesterol, LDL cholesterol, and triglycerides, were analyzed in 15 healthy adults [27]. This study was conducted in healthy participants using calcium alginate extract (5 g). The participants consumed noodles containing calcium alginate extract. Calcium-alginic acid (Ca-Alg) was used to evaluate the effects of postprandial (2 h) dyslipidemia biomarkers. However, a significant association between calcium alginate extract and hyperlipidemia biomarkers was not observed in the healthy participants. The effect of calcium alginate extract in lowering dyslipidemia biomarkers, such as total cholesterol (MD −1.070, 95% CI −4.580 to 2.440), HDL cholesterol (MD −0.400, 95% CI −1.408 to 0.608), LDL cholesterol (MD −0.070, 95% CI −2.139 to 1.999), and triglycerides (MD −0.860, 95% CI −5.809 to 7.529), was insignificant. The results indicated that a dose of 5 g calcium alginate did not have a significant effect on lowering dyslipidemia biomarker levels in healthy participants.

#### 3.5.5. Evidence of Fucoxanthin

Mikami et al. (2017) analyzed the effect of fucoxanthin extract (220 mg) on dyslipidemia biomarkers in individuals with normal weight/obesity [34]. The participants were administered a capsule of fucoxanthin-enriched akamoku oil daily for eight weeks. Each capsule appeared identical, but contained different amounts of fucoxanthin akamoku oil (0 and 220 mg). The participants were asked to continue a normal diet and physical activity, along with intake of one capsule daily after dinner for eight weeks. The consumption of 0 mg capsules, without akamoku oil, by the control group indicated insignificant effects on HDL cholesterol (MD 2.000, 95% CI 0.247 to 3.753) and triglyceride levels (MD 106.000, 95% CI 92.513 to 119.487), whereas the consumption of fucoxanthin extract significantly lowered total cholesterol (MD −9.000, 95% CI −14.612 to −3.388) and LDL cholesterol levels (MD −12.000, 95% CI −17.130 to −6.870). In this study, fucoxanthin extract (220 mg) significantly affected dyslipidemia biomarkers in normal weight and obese participants.

### 3.6. Risk of Bias Assessment

**Figure 6:**
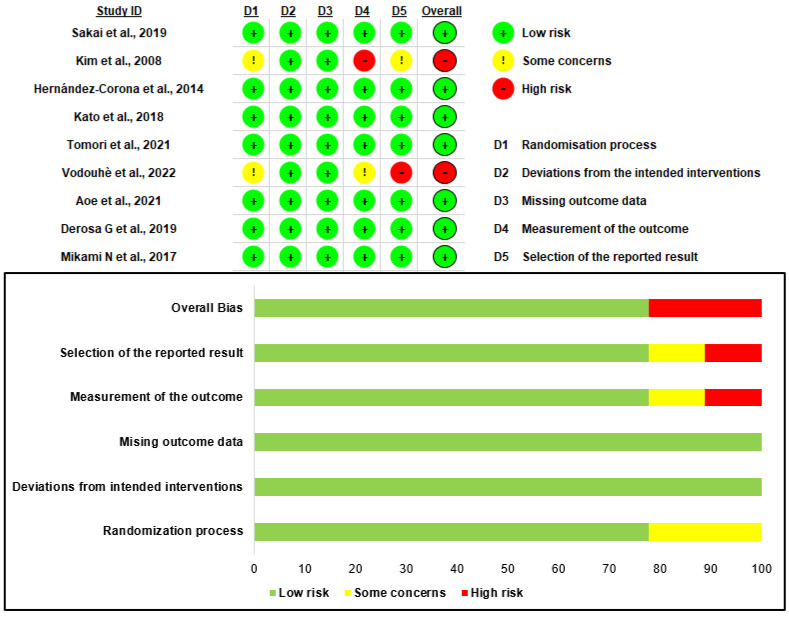
Result of the risk of bias assessment. Refs are [13,27,28,29,30,31,32,33,34].

Figure 6 depicts the risk of bias in the nine selected studies. As all studies were randomized controlled trials, the quality of the studies was evaluated using RoB 2.0. In the randomization process section, seven studies were classified as low risk, in the deviations from the intended intervention section, all studies were classified as low risk, and all studies were designated as low risk in the component of the study that lacked outcome data. In the outcomes section, seven low-risk, one of some concern, and one high-risk patients were identified. In the section on reported result selection, one high risk, one of some concern, and seven low risks were identified. Based on the algorithm, the overall evaluation indicated seven low risk and two high risk cases.

### 3.7. Publication Bias

**Figure 7:**
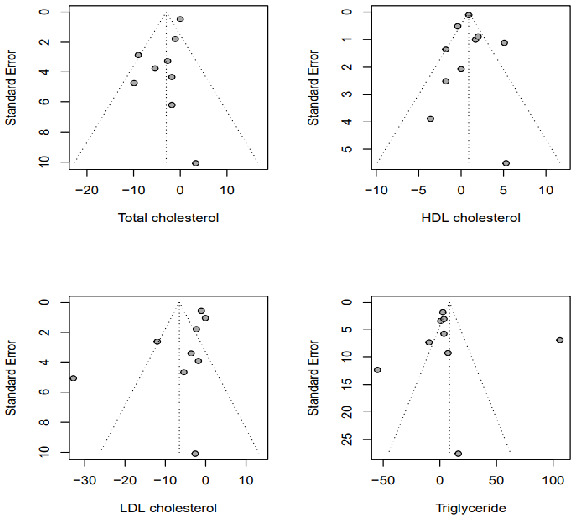
Funnel plots of publication bias.

Publication bias was evaluated using Egger’s, Begg’s, and Mazumdar’s tests. No significant publication bias was observed in the funnel plots (Figure 7). Egger’s regression coefficient test and Begg and Mazumdar’s correlation were used to calculate *p*-values to assess whether the meta-analysis contained publication bias or small-study effects. Begg’s and Egger’s tests for publication bias indicated no significant asymmetry in any of the analyses (*p* > 0.05); thus, no publication bias was observed in our analysis.

## 4. Discussion

A total of 9 randomized controlled trials with 232 participants found that brown seaweed had a positive effect on lowering biomarkers of dyslipidemia, total cholesterol, and LDL cholesterol. However, no significant association was observed between HDL cholesterol and triglyceride levels. These mixed findings may be due to differences in the type of brown seaweed or its extract, dosage, trial duration, and population characteristics.

In this meta-analysis, we found that brown seaweed intake decreased total cholesterol levels (mean difference: −3.001; 95% CI: −5.770, −0.232). In addition, significant heterogeneity was observed among the studies (*P* = 0.04; I^2^ = 51%). André et al. (2021) [18] found a hypocholesterolemic effect of brown algae species such as *Ecklonia stolonifera, Ecklonia cava, Iyengaria stellata, Colpomenia sinuosa, Spatoglossum asperum, Heterochordaria micracanthum, Sargassum patens, Cystoseria sisymbrioides, Laminaria diabolica, Sargassum ringgoldianum, Padina arborescens*, and *Undaria pinnatifida*. Furthermore, Mikami et al. (2017) found that the consumption of fucoxanthin extract significantly reduced total cholesterol levels in normal weight and obese Japanese adults [34]. At eight weeks, the same trend was observed for total cholesterol levels in non-hyperlipidemic individuals with daily kelp (*Laminaria japonica*) intake (*p* = 0.05) [29]. A male Wistar rat experiment and a randomized double-blind study of participants with mild hypercholesterolemia revealed that soluble fiber and plant sterols prevented cholesterol absorption in the gut, thereby reducing total cholesterol levels in the blood [35,36]. Dietary fiber in seaweed affects cholesterol levels, probably because of the combination of dietary fiber with bile-binding salts and other steroids in the intestine, interfering with cholesterol absorption in the jejunum and thereby increasing the excretion of cholesterol and bile acids in the feces [37,38].

Our findings demonstrated that with brown seaweed intake, HDL cholesterol levels increased, but the impact was not statistically significant in both the control and experimental groups (mean difference: 0.889; 95% CI: −0.558, 2.335). Kim et al. (2008) demonstrated that people with type 2 diabetes taking powdered sea tangles and sea mustard pills showed increased HDL cholesterol levels (*p* < 0.05) [13]. An animal study conducted in 2012 indicated that dietary fucoxanthin (0.2%) increases HDL cholesterol levels and decreases hepatic cholesterol levels in obese and diabetic mice [39]. Fucoxanthin is the most prevalent carotenoid in brown seaweed and has antioxidant properties [18,40]. A previous review indicated that the induction of sterol regulatory element-binding proteins is associated with the promotion of HDL cholesterol by fucoxanthin in brown seaweeds [41]. Fucoidan from *Laminaria japonica* elevates HDL cholesterol levels in hyperlipidemic rats (*p* < 0.05 ) [42]. Fucoidan ingestion increased HDL cholesterol levels by blocking the expression of fat synthetase genes in HepG2 hepatocytes and liver tissues [43].

A reduction in LDL cholesterol levels through brown seaweed intake was demonstrated in this analysis (mean difference: −6.519; 95% CI: −12.884, −0.154). High heterogeneity was observed in our analysis (*P* < 0.01, I^2^ = 86%). In the findings of Aoe et al. (2021), LDL cholesterol levels indicated a significant decrease (−0.17 mmol/L) in the test group who consumed iodine-reduced kelp (*Laminaria japonica*) powder at eight weeks compared with that of placebo group in non-hyperlipidemia participants [29]. A meta-analysis of 12 trials with 807 participants evaluated the effects of spirulina intake on lipid concentrations [44]. Total cholesterol and LDL cholesterol levels decreased by −1.00 mmol/L and −0.91 mmol/L, respectively, while HDL cholesterol concentration did not significantly change [44]. However, directly comparing these findings with our analysis is challenging because of the differences in seaweed types. A pharmacological experiment in experimental hyperlipidemic rats published in 2004 found that *Sargassum fusiforme* polysaccharide, a marine algae-sulfated polysaccharide containing alginic acid, fucoidan, and a small amount of laminaran and dietary fiber, decreased the levels of LDL cholesterol, total cholesterol, and triglycerides [45,46].

Since seaweed polysaccharides have the characteristics and functions of diffusing in water, retaining cholesterol and relevant physiologically active compounds, then preventing the absorption of lipids in the gastrointestinal tract, they have been shown to reduce serum cholesterol levels [47]. No significant difference was found in the effect of brown seaweed on triglyceride levels between the experimental and control groups (mean difference: 8.515; 95% CI: −19.354, 36.383). An animal experiment demonstrated that after administration of 100 mg/kg fucoidan daily for 4 weeks, triglyceride, LDL cholesterol, and total cholesterol levels in obese mice decreased by 21.09%, 6.35%, and 18.74%, respectively [48]. A previous study found that a nutritional formulation containing brown seaweed extract significantly reduced triglyceride levels in rats with nonalcoholic steatohepatitis and nonalcoholic fatty liver disease [49]. Kim et al. (2008) indicated that serum triglyceride levels decreased in patients with type 2 diabetes who consumed 48 g of seaweed daily for 4 weeks [13]. In the present analysis, only one study did not measure triglycerides. In three studies on patients with type 2 diabetes, no comparison could be made because the dose used by Derosa et al. (2019) was not mentioned [31]. The difference in the effects of lowering triglyceride levels mentioned by Kim et al. (2008) and Sakai et al. (2019) could be due to dosage differences in seaweed [13,30]. The total daily consumption of seaweed reported by Kim et al. (2008) was 48 g, which was higher than that reported by Sakai (1620 g). As a natural supplement, seaweeds, especially brown seaweeds, can be used in combination with antihyperlipidemic drugs, such as statins and fibrates [50,51,52]. A potential approach for incorporating brown seaweed into nanoparticles for improved tissue-targeted delivery has also been introduced [53].

This study has several strengths. To our knowledge, this is the first study to examine the effects of brown seaweed on dyslipidemia biomarkers in humans. Second, we considered the varieties of brown seaweeds, such as fucoidan extract, *Laminaria japonica*, and calcium alginate extract to perform a systematic review of meta-analyses on brown seaweed consumption and biomarkers of dyslipidemia. Despite these strengths, this study has several limitations. First, we could not examine the dose–response association between brown seaweed and dyslipidemia biomarkers because the types of brown seaweed varied across studies. Second, as most studies on brown seaweed in dyslipidemia have been conducted on animals, this systematic review and meta-analysis included only a limited number of human studies.

## 5. Conclusions

This systematic review and meta-analysis imply that brown seaweed and its extracts may reduce the risk of dyslipidemia. A meta-analysis indicated that brown seaweed and its extracts are significantly associated with lower total cholesterol and LDL cholesterol levels. Our findings have important implications and potential benefits for patients with dyslipidemia. Further studies involving a larger population are necessary to investigate the dose–response association between brown seaweed and dyslipidemia.

## Figures and Tables

**Table 1:** PICOS criteria for inclusion of studies.

Parameter	Criterion
Population	Healthy participants, participants with pre-diabetes or type 2 diabetes mellitus, participants with obesity
Intervention	Mozuku fucoidan extract, *Laminaria japonica* (Sea tangle), Fucoidan extract, Sodium alginate extract, Fucoidan derived from Okinawa mozuku
Comparison	Placebo
Outcome	Total cholesterol, HDL cholesterol, LDL cholesterol, triglycerides
Study design	Randomized controlled trials, clinical trials

**Table 3:** Results of the meta-regression analysis.

	Total Cholesterol			HDL Cholesterol			LDL Cholesterol			Triglyceride		
Variation	*k*	SMD (95% CI)	*p*	*k*	SMD (95% CI)	*p*	*k*	SMD (95% CI)	*p*	*k*	SMD (95% CI)	*p*
**Duration**			0.598			0.172			0.128			0.666
>3 months	5	−2.711 (−7.991–2.570)		4	0.027 (−2.623–2.677)		5	−3.144 (−11.885–5.597)		5	3.633 (−39.159–46.425)	
<3 months	3	−4.681 (−9.755–0.392)		3	2.461 (0.184–4.737)		3	−13.775 (−24.314–−3.236)		3	18.845 (−35.305–72.995)	
**Race**			0.937			0.327			0.738			0.919
Asian	5	−4.032 (−8.852–0.788		5	2.057 (−0.193–4.307)		5	−10.118 (−20.527–0.291)		5	15.116 (−31.827–62.058)	
Hispanic	1	−1.802 (−13.130–9.527)		-	-	-	1	−5.405 (−28.578–17.767)		1	7.207 (−96.122–110.536)	
Caucasian	2	−3.968 (−11.163–3.226)		2	0.083 (−3.164–3.330)		2	−2.930 (−18.448–12.588)		2	−2.895 (−75.240–69.451)	
**Female** **participants rate**	8	−13.141 ^a^ (−42.559–16.316)	0.382	7	5.994 ^a^ (−13.116–25.104)	0.539	8	−14.172 ^a^ (−70.258–41.914)	0.620	8	86.767 ^a^ (−153.590–327.124)	0.479

k, number of effect sizes; CI, confidence interval; SMD, standardized mean difference. *p*-value obtained from meta-regression analysis using restricted maximum likelihood. Meta-regression analysis was performed for continuous variables (female participation rate), and meta-ANOVA analysis was performed for categorical variables (duration, race/ethnicity). ^a^, regression coefficient.

## Data Availability

Data supporting the findings of this study are available from the corresponding author upon reasonable request.

## Supplementary Materials

The following supporting information can be downloaded at: https://www.mdpi.com/article/10.3390/md21040220/s1, Table S1: Search queries.
