# Supplementary material for: How Do Brown Seaweeds Work on Biomarkers of Dyslipidemia? A Systematic Review with Meta-Analysis and Meta-Regression
Source: Mar Drugs. 2023 Mar 30;21(4):220. doi: 10.3390/md21040220 (PMC10144066; doi:10.3390/md21040220)
Supplement: Supplementary file 1 [file marinedrugs-21-00220-s001.zip › marinedrugs-2251004-supplementary.pdf]

Supplementary materials

Table S1. Search queries.

PubMed and Cochrane Library

("Seaweed"[Mesh] OR "Seaweed"[tiab] OR "Brown Seaweed"[tiab] OR "Brown Seaweed Extract"[tiab] OR "Phaeophyta"[Mesh] OR "Phaeophyta"[tiab] OR "Brown Algae"[tiab] OR "Phaeophyceae"[tiab] OR "Brown Algae Extract"[tiab] OR "fucoidan" [Supplementary Concept] OR "fucoidan" [tiab] OR "ventol" [Supplementary Concept] OR "dioxinodehydroeckol" [Supplementary Concept] OR "2,7"-phloroglucinol-6,6'-bieckol" [Supplementary Concept] OR "Ecklonia cava"[tiab] OR "Undaria"[Mesh] OR "Undaria"[tiab] OR "Wakame"[tiab] OR "fucoxanthin"[Supplementary Concept] OR "fucoxanthin"[tiab]) AND ("Cholesterol"[Mesh] OR Cholesterol[tiab] OR Epicholesterol[tiab] OR "HDL Cholesterol"[tiab] OR "High Density Lipoprotein Cholesterol"[tiab] OR "Dyslipidemias"[Mesh] OR "Total-cholesterol"[tiab] OR "Total cholesterol"[tiab] OR "Triglycerides"[Mesh] OR "Triglyceride"[tiab]) AND ((clinicaltrial[Filter]) AND (humans[Filter]))

Embase

('seaweed'/exp OR 'seaweed':ti,ab OR 'brown seaweed':ti,ab OR 'brown seaweed extract':ti,ab OR 'brown alga'/exp OR 'phaeophyta':ti,ab OR 'brown algae':ti,ab OR 'phaeophyceae':ti,ab OR 'brown algae extract':ti,ab OR 'undaria'/exp 'ecklonia cava'/exp OR 'dioxinodehydroeckol'/exp OR 'salbutamol'/exp OR 'fucoidin'/exp OR 'fucoidan':ti,ab OR 'fucoidin':ti,ab OR 'ventol':ti,ab OR 'dioxinodehydroeckol':ti,ab OR 'ecklonia cava':ti,ab OR 'undaria':ti,ab OR 'wakame':ti,ab) AND ('human'/de) AND ('clinical trial'/de OR 'controlled clinical trial'/de OR 'controlled study'/de OR 'double blind procedure'/de OR 'randomized controlled trial'/de OR 'major clinical study'/de)
